# Palaeodietary traits of large mammals from the middle Miocene of Gračanica (Bugojno Basin, Bosnia-Herzegovina)

**DOI:** 10.1007/s12549-020-00435-2

**Published:** 2020-06-11

**Authors:** Alexandros Xafis, Juha Saarinen, Katharina Bastl, Doris Nagel, Friðgeir Grímsson

**Affiliations:** 1 Department of Palaeontology, Faculty of Earth Sciences, University of Vienna, 1090 Vienna, Austria; 2 Department of Geosciences and Geography, University of Helsinki, Helsinki, Finland; 3 Department of Oto-Rhino-Laryngology, Research Group Aerobiology and Pollen Information, Medical University of Vienna, Vienna, Austria; 4 Department of Botany and Biodiversity Research, University of Vienna, 1030 Vienna, Austria

**Keywords:** Dental wear, Microwear, Mesowear, Palaeoecology, Feeding adaptation, Palaeodiet

## Abstract

Recent excavations at the Gračanica coal mine (Bugojno Basin, Bosnia-Herzegovina) have unearthed numerous skeletal parts of fossil vertebrates, including a noteworthy collection of mammalian remains. Previous palaeoecological investigations of the Dinarides Lake System were established using stratigraphical, palaeofloral, and malacological data. However, large mammal remains have so far not been used to reconstruct the terrestrial palaeoenvironment of this important fossil ecosystem. Here, the palaeodietary preferences of large mammals were investigated, using a multiproxy approach by employing dental microwear and dental mesowear analysis, in order to provide new perspectives on the terrestrial palaeoecology of the Dinarides Lake System. The dental microwear of all available adult mammalian teeth was analysed. Dental mesowear analysis was employed for ungulate and proboscidean taxa, using mesowear scores and mesowear angles, respectively. The analysis reveals the presence of browsing, “dirty browsing”, and mixed-feeding herbivorous taxa, with seasonal fruit, or even grass intake. Additionally, the analysis of the carnivores suggests the presence of hyaena- and cheetah-like hypercarnivores, as well as generalists. The palaeodietary traits of the fossil mammals suggest a closed canopy-like environment, which is supported by the fossil plant assemblage. Palaeopalynological data confirm the omnipresence of fleshy fruit-bearing plants, herbaceous taxa, as well as grasses, which justifies the seasonal fruit browsing, the common “dirty browsing”, and the occasional grazing behaviour visualized for some of the fossil mammals from Gračanica.

**Electronic supplementary material:**

The online version of this article (10.1007/s12549-020-00435-2) contains supplementary material, which is available to authorized users.

## Introduction

Recent excavations at the Gračanica coal mine (Bugojno Basin, Bosnia-Herzegovina), conducted by the Natural History Museum of Vienna, Department of Palaeontology, have unearthed numerous skeletal parts of fossil vertebrates. These, combined with several fossils from local collectors, yield a noteworthy collection of vertebrate remains.

**Fig. 1:**
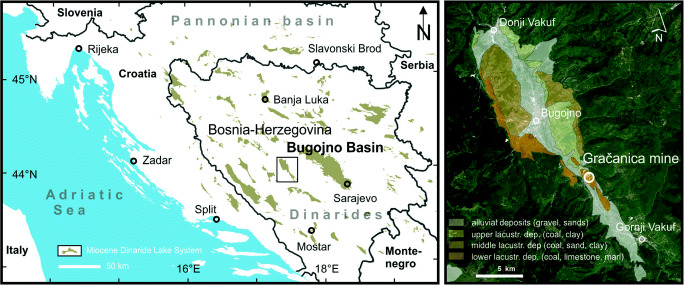
Geographic position of the fossiliferous locality at Gračanica (Bosnia-Herzegovina) (left) and geological map of the Bugojno Basin (right) (after Mandic et al. 2012, 2016)

The Gračanica coal mine is situated near the village of Gračanica, approximately 10 km SSE of the centre of Bugojno (Fig. 1). The fossiliferous locality is part of the intramontane Bugojno Basin, which is filled with Neogene lacustrine and alluvial sediments. The sedimentary succession consists of three deposition cycles (Čičić 1976). In the Gračanica pit, the lowermost cycle rests on the Permian and Triassic carbonate and siliciclastic basement (Mandic et al. 2016). This cycle consists in its lower part of numerous recurrent coal seams, marls, siltstones, and limestones, reflecting a wetland palaeoenvironment (Mandic et al. 2016, Mandic et al. in press, this issue; Jimémez-Moreno and Mandic in press, this issue). The Gračanica coal mine was initially exploited in 1939 and since then, numerous vertebrate remains have been discovered (Mandic et al. 2016; Mandic et al. in prep.). The vertebrate remains reveal a plethora of taxa, including 29 mammalian and at least six non-mammalian species. The fossil mammals are represented by soricids, insectivores, rodents, ruminants, suids, rhinocerotids, chalicotheriids, equids, proboscideans, and carnivores, the taxonomic attribution of which is presented throughout this special issue (Wessels et al. in press, this issue; Stefen in press, this issue; Aiglstorfer and Mayda in press, this issue; Van der Made in press., this issue; Becker and Tissier in press, this issue; Coombs and Göhlich in press, this issue; Becker et al. in press, this issue; Göhlich in press., this issue; Bastl et al. in press, this issue).

The Gračanica deposits are of middle Miocene age. The small mammal fossil assemblage points to the early middle Miocene, between the Mammal Neogene Units MN4-MN6 (Wessels et al. in press, this issue). The Suidea suggest an age between 14.05 and 13.7 Ma (Van der Made in press, this issue). The endemic lacustrine mollusks from the Gracanica section point to an age slightly younger than 15 Ma (Mandic et al. in press.). Correlation of the pollen palaeoclimate proxy with orbital parameters and insolation points to an age of the Gračanica section between 14.80 and 14.55 Ma (Jimémez-Moreno and Mandic in press, this issue).

This rich and diverse fossil mammal assemblage is highly important for reconstructing the middle Miocene palaeoenvironment of the western Dinarides. To date, palaeoecological investigations of the Dinarides Lake System (DLS) were established by stratigraphical, palaeofloral, and malacological data (De Leeuw et al. 2010, 2011, 2012; Harzhauser and Mandic 2008, 2010; Jiménez-Moreno et al. 2008, 2009; Mandic et al. 2009, 2011; Neubauer et al. 2011, 2013; Sant et al. 2018). Therefore, this investigation on the dietary behaviour of large mammals provides a completely new perspective on the terrestrial palaeoecology of the DLS. In addition, it presents the first data on mammalian palaeodietary preferences in the western part of the Dinarides-Anatolian Island, which formed a terrestrial barrier between the Paratethys and the proto-Mediterranean Sea (or Mediterranean Tethys) during the early and middle Miocene (Mandic et al. 2011; Rögl 1998).

During the last four decades, palaeoecologists have established various dietary proxies, with dental microwear (Grine 1977; Rensberger 1978; Walker et al. 1978; Solounias and Semprebon 2002; Ungar et al. 2003; Scott et al. 2006; Kaiser and Brinkmann 2006; Merceron 2003; Merceron et al. 2004; Merceron et al. 2005a; Semprebon et al. 2004a) and dental mesowear (Fortelius and Solounias 2000; Kaiser and Solounias 2003; Schubert 2007; Mihlbachler et al. 2011; Solounias et al. 2014; Fraser et al. 2014) being the two most widely used. Dental microwear is defined as the result of the abrasion of a tooth surface, due to food particles and represents the “last supper” of an animal (Grine 1986; Mainland 1998; Solounias et al. 1988; Teaford and Oyen 1989; Fortelius and Solounias 2000; Merceron et al. 2004). Dental mesowear is the result of abrasion and attrition (defined as the tooth-on-tooth wear), the magnitude of which is responsible for the relief and sharpness of cusp apices in ungulates (Fortelius and Solounias 2000; Rivals et al. 2018). Mesowear exhibits a deeper time perspective on the dietary ecology of an animal, since it reflects the cumulative wear during an animal’s lifetime (Semprebon et al. 2016). For almost two decades, mesowear was exclusively applied to ungulates, due to the similar cusp morphology. However, recent studies have shown that mesowear can also be applied to other mammal groups such as the small Leporinae and Murinae (Ulbricht et al. 2015), as well as the much larger Proboscidea (Saarinen et al. 2015).

Dental microwear features can be observed on the enamel surface and are classified into three main categories (pits, scratches, and gouges), which are further subdivided based on their size and light refractive properties (Semprebon et al. 2004a). More specifically, pits are defined as mostly circular microwear scars, and according to their size, they can be distinguished into small (bright and highly refractive), large (dark and less refractive), and puncture pits (very large and crater-shaped). Similarly, scratches are elongated features and subdivided into fine (narrow lines, averagely refractive), coarse (wide and deep lines, highly refractive), and hypercoarse scratches (very deep and wide, less refractive). Gouges are irregular features, which are larger and deeper than large pits and generally less refractive. The number of pits and scratches, as well as the presence or absence of gouges, can discriminate between different dietary categories of herbivorous and carnivorous taxa (Solounias and Semprebon 2002; Bastl et al. 2012).

Herbivorous taxa are placed into three fundamental dietary categories based on their food preferences: browsers, grazers, and mixed feeders (Hofmann 1989). Since grasslands evolved only during the late Cenozoic (Janis 2008), browsing constitutes the more plesiomorphic feeding type. Under the microscope, leaf browsers have a larger variation of pits on the enamel surfaces, while grazers exhibit a larger amount of scratches, as well as large pits, as a result of the more extensive quantity of phytolith and exogenous dust and grit intake (Winkler et al. 2019; Ackermans et al. 2020). Mixed-feeding taxa fall in between the two main dietary categories because of seasonal and/or regional variation in food preferences (Walker et al. 1978; Solounias and Hayek 1993; Solounias and Semprebon 2002; Merceron et al. 2004; Merceron et al. 2005b). In addition, frugivores (or fruit browsers) are taxa that incorporate a significant amount of fruits and seeds in their diet, which results in a more dominant presence of coarse scratches, large pits, and puncture pits. Macroscopically, a less abrasive and more attritive diet results in sharpened apices on the buccal cusps. Contrariwise, a more abrasive (less attritive) diet results in more flattened and rounded buccal cusp apices (Fortelius and Solounias 2000).

The dietary traits of the order Carnivora are much more complex and diversified, containing a large variety of nutritional components, such as meat, bone, plant fibre, fruit, eggs, and insects (Wilson and Mittermeier 2009). Diets of Carnivora can be classified into three main categories based on the amount of meat intake: hypocarnivorous, mesocarnivorous, and hypercarnivorous. These three groups include a large variety of specialised consumers, such as piscivores, herbivores, larvae, and worm eaters (Sacco and Van Valkenburgh 2004; Goillot et al. 2009). The hypercarnivorous diet is further differentiated into cat-like, hyaena-like, and wolf-like ecomorphs (Van Valkenburgh 1988; Wesley-Hunt 2005; Van Valkenburgh 2007). Similar to herbivorous taxa, Carnivora with a large herbal intake exhibit a high number of scratches on the enamel surfaces of their teeth, the rest of the dietary groups do not show any noteworthy differences in the amount of scratches or pits (Goillot et al. 2009).

This study constitutes the first palaeodietary investigation of an early-middle Miocene European locality utilising fossil premolars and non-carnassials in a low-magnification microwear analysis. Our main objective was to reconstruct dietary traits of all large mammalian dental remains from Gračanica. To achieve this, we performed a multiproxy analysis, utilising the methods of dental microwear and dental mesowear analyses. Microwear analysis was conducted on both herbivorous and carnivorous taxa. Dental mesowear analysis was employed on ungulate and proboscidean taxa. As a working hypothesis, and based on the available geological and palaeontological data, we expect that the dietary traits of the Gračanica fossil assemblage will reflect a closed forest environment. If our hypothesis is correct, we expect that the palaeocommunity was dominated by browsing taxa and that pure grazers were absent.

## Anatomical and institutional abbreviations

P2/p2: upper/lower second premolar; P3/p3: upper/lower third premolar; P4/p4: upper/lower fourth premolar; M1/m1: upper/lower first molar; M2/m2: upper/lower second molar; M3/m3: upper/lower third molar; NHMW: Fossil Mammal Collection, Naturhistorisches Museum Wien (Vienna, Austria); NMW: Zoological Collection, Naturhistorisches Museum Wien (Vienna, Austria); ZMB_Mam: Zoological Mammal Collection, Museum für Naturkunde, Berlin (Berlin, Germany); RBINS: Royal Belgian Institute of Natural Sciences (Brussels, Belgium); NHML: Fossil Mammal Collection, Natural History Museum (London, United Kingdom) MGM: Museo Geominero (Madrid, Spain); HLMD: Hessisches Landesmuseum (Darmstadt, Germany).

## Materials and methods

The Gračanica fossil dental material incorporated in this study is stored in the collection of the NHMW. All specimens from large mammalian groups including Cetartiodactyla, Perissodactyla, Proboscidea, and Carnivora were available for this study. Table 1 shows the taxa used for each analysis.

**Table 1:** List of mammalian taxa from Gračanica considered for dental microwear and mesowear analyses. Note: (+) indicates incorporation of taxon in analysis; (−) indicates exclusion of taxon from analysis

Group	Taxon	Microwear	Mesowear
Ruminantia	*Dorcatherium vindebonense*	+	−
	Palaeomerycidae indet.	+	+
	*Giraffokeryx* sp.	+	−
	?Tethytragus sp.	+	−
	*Eotragus ?clavatus*	+	+
Suoidea	*Choeromorus lemuroides*	+	−
	*Bunolistriodon latidens*	+	−
	*Conohyus simorrensis*	+	−
Rhinocerotidae	*Brachypotherium brachypus*	+	+
	*Hispanotherium* cf. *matritense*	+	−
	*Lartetotherium sansaniense*	+	−
	*Plesiaceratherium balkanicus*	+	+
Equidae	*Anchitherium ezquerrae*	+	+
	*Anchitherium hippoides*	+	+
Chalicotheriidae	*Anisodon* cf. *grande*	−	+
Proboscidea	*Prodeinotherium bavaricum*	+	+
	*Gomphotherium angustidens*	+	+
	cf. *Protanancus* sp.	−	−
	cf. *Gomphotherium subtapiroideum*	−	−
Carnivora	*Amphicyon giganteus*	+	−
	*Hemicyon goeriachensis*	+	−
	*Ursavus brevirhinus*	+	−
	*Percrocuta miocenica*	+	−
	Mustelidae indet.	−	−

For the evaluation of microwear and mesowear data obtained from the Gračanica fossils, four extant comparative datasets were used, which were created based on potential modern analogues. For microwear analysis of herbivorous taxa, an updated dataset by Xafis et al. (2017) was used. Since the latter included only ruminant artiodactyls, the dataset was enriched with additional ungulate, as well as proboscidean taxa. The new data are based on specimens housed in the collections of ZMB_Mam, NMW, and RBINS. The updated dataset includes 282 entries and is presented in the Online Resource 1. For microwear analysis of Carnivora, the microwear dataset of Xafis et al. (2017: updated from Bastl et al. 2012) was employed. For mesowear analysis of ungulate taxa, a new comparative dataset was created. The extant mesowear dataset consists of 43 ungulate taxa, including 453 entries, covering all herbivorous dietary categories (Online Resource 2). For dental mesowear analysis of proboscidean taxa, the comparative dataset including fossil and extant taxa is presented in Online Resource 3.

### Microwear analysis

Since it has been shown that all post canine teeth of ruminants and carnivores exhibit insignificant differences in their microwear signals, we follow the sampling procedure of Xafis et al. (2017). Thus, premolars and molars, as well as non-carnassials and carnassials, were included in our microwear analysis. All teeth were prepared following the general protocol established by Grine (1986). The cusps were soaked in acetone using cotton patches and foam swabs, to remove shellac, glue, or other preparation adhesives. After 20 to 30 min, the same areas were wiped with 95% ethanol. When the surfaces were dry, the teeth were moulded, using a high-consistency and high-precision polyvinylsiloxane dental impression material. After some minutes, the dry moulding material was removed from the teeth and hemmed in a thin layer of two-component polysiloxane lab putty, to form a cup-like wall around the sample. Subsequently, the form was filled with a clear epoxy resin, which was centrifuged prior to application, in order to extract bubbles out of the liquid and the cast. Following the general methodology of low-magnification microwear analysis by Solounias and Semprebon (2002) and Semprebon et al. (2004b), the epoxy casts were examined at 35× magnification within a standardized area of 0.4 × 0.4 = 0.16 mm^2^ using a Leica MZ12 stereomicroscope and a Leica CLS 100 oblique lighting source.

**Fig. 2:**
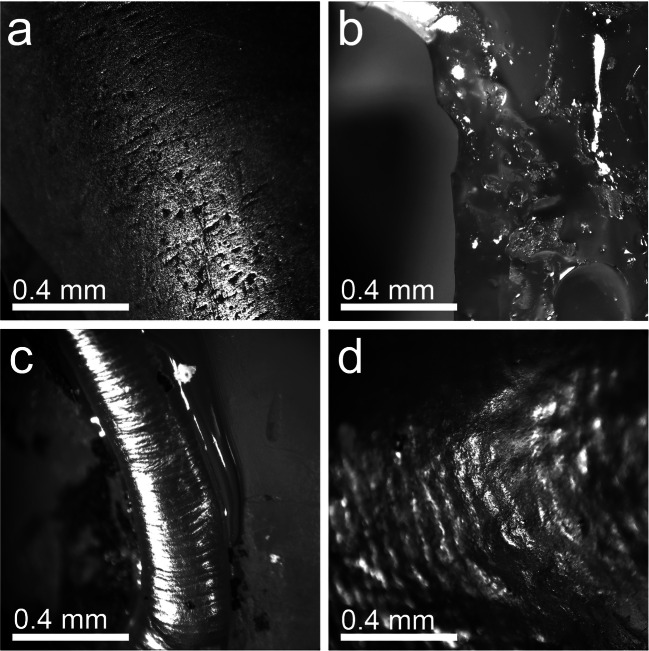
Photomicrographs of shearing facets, displaying four non-microwear features detected during screening. **a** Taphonomic alteration on *Prodeinotherium bavaricum*, **b** adhesive coating on Mustelidae indet., **c** Hunter-Schregger bands on *Brachypotherium brachypus*, and **d** perikymata on *Bunolistriodon latidens*. All images represent × 35 magnification; scale bar equals 0.4 mm

Prior to the analysis of the casts, a screening was performed following Hunter and Fortelius (1994), King et al. (1999), and El-Zaatari (2010) to detect natural and/or artificial non-microwear features, caused by taphonomic processes or preparation techniques (Fig. 2). Due to the differences in tooth morphology and topography, two experienced observers were employed to analyse the epoxy casts, to guarantee consistent results: all herbivore samples were counted by AXE and all Carnivora specimens were counted by KB. The extant comparative microwear datasets used for the evaluation of herbivore and carnivore taxa were created by the respective observers (see Online Resource 1 and Xafis et al. 2017: Table S2.)

### Mesowear analysis

In the ungulate taxa, dental mesowear was scored macroscopically from the buccal side of maxillae or isolated upper molars and the lingual side of mandibles or isolated lower molars. Following Fortelius and Solounias et al. (2000), the paracone of the M2 was mostly sampled. Additionally, because of the scarcity of the fossil material, we included m1, m2, m3, M1, and M3 in our analysis to increase the sample size. Based on Rivals et al. (2007), a screening was performed taking into consideration the age of the individuals, as well as the condition of the dental elements. Therefore, unworn or slightly worn, extremely worn, and damaged teeth were excluded from the analysis. For the mesowear stage scoring, the standardized method of Mihlbachler et al. (2011) was used. Since the two originally proposed measurements, cusp sharpness and relief, are not independent from each other, seven cusp divisions (from 0 to 6) were introduced. Category 0 represents very sharp cusps with high relief and category 6 depicts completely flat cusps with no relief (Mihlbachler et al. 2011). The average number of mesowear values from the dentition of each taxon represents the mesowear score (MWS) (Mihlbachler et al. 2011).

Mesowear angle analysis (Saarinen et al. 2015) was used for the dietary analyses of the Gračanica proboscidean specimens. The method is based on measuring the relief of worn enamel ridges on the occlusal surface of worn molar teeth as angle measurements, by fitting the tip of an angle at the bottom of worn dentine valleys within lophs/lamellae on the molar surface and the sides of the angle as tangent to the top of the enamel ridges. A digital angle metre with added movable plates (Saarinen et al. 2015) was used for measuring the angles at 0.1° precision. The mesowear angles were measured from equivalent locations for each proboscidean molar tooth type:

1. For molars with continuous transverse lophs or lamellae, with dentine valleys forming as a result of wear, the mesowear angles were measured from the middle of the lamellae at the centre of the tooth (Saarinen et al. 2015).
2. For molars with more bunodont morphology, the mesowear angles were measured from the deepest worn dentine valley within each transverse pair of cusps (or “loph”).
3. In cases where worn dentine valleys had not developed as a result of tooth wear, but where wear facets had formed in the enamel, the mesowear angles were measured from the wear facets by placing one side of the angle parallel to the facet and the other parallel to the level of the occlusal surface (angle A), and then calculating the mesowear angle (B) as: B = 180° − (2 × [180° − A]).

The mesowear angles of the Gračanica proboscideans were compared with those of other palaeopopulations of Miocene proboscideans from Eurasia, and representative extant populations of African forest elephant (*Loxodonta cyclotis*), African savanna elephant (*Loxodonta africana*), and Asian elephant (*Elephas maximus*) by pairwise Wilcoxon tests. The comparative proboscidean material comes from the collections of the NHML, MGM, and HLMD (Online Resource 3).

Due to the difference in the collection of MWS and the measuring of mesowear angles for ungulates and proboscideans, respectively, the data were collected by two investigators. Dental mesowear scores were collected by AX and mesowear angles were measured by JS. Even though dental mesowear exhibits an insignificant interobserver error (Loffredo and DeSantis 2014), in order to eliminate any sample bias, the extant mesowear comparative datasets used in this study were created by the respective researcher (Online Resource 2; Online Resource 3).

## Results

**Fig. 3:**
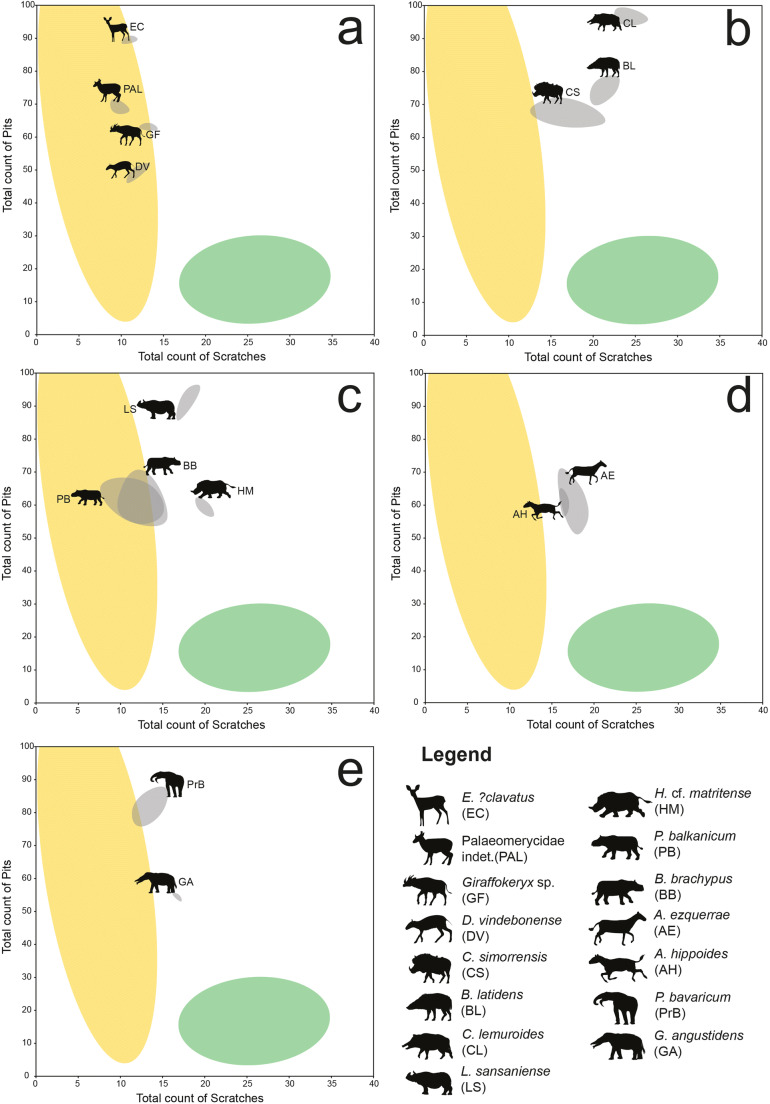
Dental microwear results of fossil herbivores from Gračanica, plotted on Total count of Scratches versus Total count of Pits scatter plots. Every graph represents a different group: **a** Ruminantia, **b** Suidae, **c** Rhinocerotidae, **d** Equidae, and **e** Proboscidea. Grey ellipses represent the distribution of each fossil taxon. Yellow and green highlighted areas represent the Gaussian confidence ellipses (*p* = 0.95) of extant browsers and grazers (after Solounias and Semprebon 2002 and data presented herein). For identification of taxa, consult the symbol legend. Animal silhouettes from PhyloPic.org

**Fig. 4:**
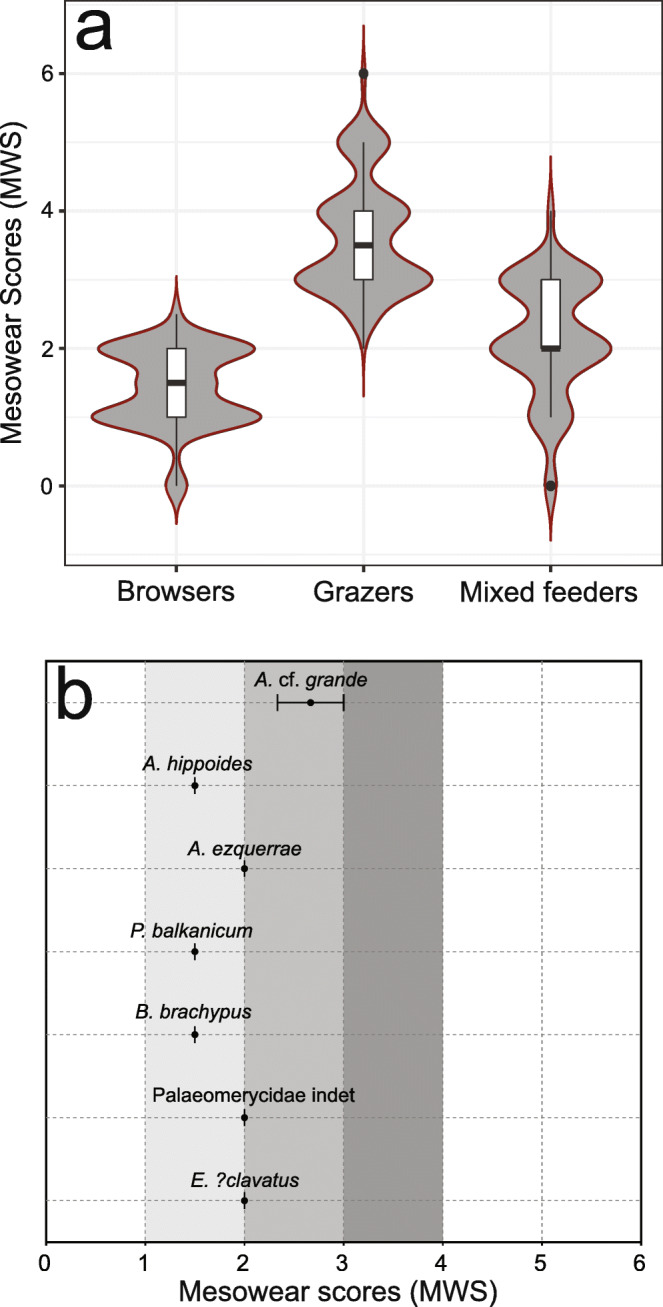
**a** Violin plot presenting the MWS of all ungulates (browsers, grazer, and mixed feeders) included in the extant comparative dataset. Each box represents 50% of the range of the MWS, while the top and bottom whiskers represent the overall range of the MWS. The horizontal lines in the boxes represent the median sample for each dietary group. Raw data are provided in Online Resource 2. **b** Whisker plot showcasing the range of MWS for the fossil herbivores from Gračanica. Light grey area represents the interquartile range (IQR) of the extant browsers, medium grey area represents the IQR of extant mixed-feeders, and dark grey area represents the IQR of extant grazers. Whiskers demonstrate the overall range of the MWS for each taxon

The microwear results of the newly added taxa in the extant comparative dataset are presented in Online Resource 1. The dataset of Xafis et al. (2017) was enriched with postcanine teeth of 23 species from the groups Equidae, Proboscidea, Rhinocerotidae, Suidae, Tayassuidae, and Tapiridae. As expected, browsing taxa occupied the left part of the classic scratches-versus-pits scatter plot, grazing taxa clustered on the bottom right part, while generalists distributed all over the graph. The browsing and grazing morphospaces are visualized in Fig. 3, based on the new dataset, as well as the original data presented in Solounias and Semprebon (2002). The comparative mesowear dataset consists of 453 entries, belonging to 43 ruminant taxa from eight families (Antilocapridae, Bovidae, Cervidae, Equidae, Giraffidae, Moschidae, Rhinocerotidae, and Tragulidae; Online Resource 2). The results show a clear division between browsers, grazers, and generalists: browsing taxa (*n* = 153) exhibited MWS between 0.0 and 2.5 with the interquartile range or (IQR) fluctuating between 1.0 and 2.0; grazing taxa (*n* = 203) scored between 2.5 and 6.0, with the IQR fluctuation between 3.0 and 4.0; mixed-feeding taxa (*n* = 97) scored between 0.0 and 4.0 with the IQR fluctuating between 2.0 and 3.0 (Fig. 4). The microwear results of the fossil herbivores and carnivores from Gračanica are summarised in Tables 2 and 3. The raw data are demonstrated in Online Resource 4 and Online Resource 5. The dental mesowear results are presented in Table 4.

**Table 2:** Microwear results of fossil ungulates from Gračanica

Taxon	*N*	P	SE-P	S	SE-S	S/P	%F	%C	%M	%LP	%PP	%G	%0–17 S
*Dorcatherium vindebonense*	2	49.50	2.50	12.00	1.00	0.24	0.00	0.00	100.00	50.00	100.00	0.00	100.00
*Eotragus?clavatus*	2	89.00	0.50	11.00	0.50	0.12	0.00	0.00	100.00	50.00	50.00	0.00	100.00
*Giraffokeryx sp.*	2	63.25	0.25	13.25	0.75	0.21	0.00	0.00	100.00	50.00	100.00	50.00	100.00
Palaeomerycidae indet.	3	69.00	1.00	9.67	0.67	0.14	33.33	0.00	66.66	100.00	100.00	66.66	100.00
*Conohyus simorrensis*	5	66.40	1.12	17.60	1.72	0.27	0.00	0.00	100.00	100.00	100.00	100.00	40.00
*Choeromorus lemuroides*	3	96.67	0.67	24.00	1.00	0.25	0.00	0.00	100.00	100.00	100.00	100.00	0.00
*Bunolistriodon latidens*	9	73.78	0.83	21.22	0.43	0.29	0.00	0.00	100.00	100.00	66.66	100.00	0.00
*Brachypotherium brachypus*	7	59.79	2.10	12.29	0.91	0.21	0.00	0.00	100.00	100.00	100.00	42.80	100.00
*Hispanotherium* cf. *matritense*	4	59.13	1.20	20.00	0.58	0.34	0.00	0.00	100.00	100.00	100.00	0.00	0.00
*Lartetotherium sansaniense*	2	91.00	5.00	18.00	1.00	0.20	0.00	0.00	100.00	100.00	100.00	0.00	50.00
*Plesiaceratherium balkanicum*	7	59.79	1.50	12.36	0.88	0.21	28.60	0.00	71.40	100.00	100.00	0.00	100.00
*Anchitherium ezquerrae*	9	62.61	2.11	17.56	0.37	0.28	0.00	0.00	100.00	100.00	88.88	55.50	44.44
*Anchitherium hippoides*	6	61.58	1.27	16.33	0.17	0.27	0.00	0.00	100.00	100.00	100.00	16.70	100.00
*Gomphotherium angustidens*	2	54.50	1.00	16.75	0.25	0.31	0.00	0.00	100.00	100.00	50.00	0.00	100.00
*Prodeinotherium bavaricum*	8	81.56	1.18	13.69	0.57	0.17	25.00	0.00	75.00	87.50	75.00	0.00	100.00

Note: N, sample size; P, average amount of pits; SE-P, standard error of pits; S, average amount of scratches; SE-S, standard error of scratches; S/P, scratch/pit ratio; %F, percentage of individuals per taxon with fine scratches; %C, percentage of individuals per taxon with coarse and hypercoarse scratches; %M, percentage of individuals per taxon with both fine and coarse scratches; %LP, percentage of individuals per taxon with more than four large pits; %PP, percentage of individuals per taxon with puncture pits; %G, percentage of individuals per taxon with gouges; %0–17 S, percentage of individuals per taxon with 0–17 scratches

**Table 3:** Microwear results of fossil carnivores from Gračanica

Taxon	*N*	SP	SE-SP	LP	SE-LP	SP/LP	%F	%C	%M	%PP	%G	%0–8 LP
*Ursavus brevirhinus*	1	100.50	–	21.00	–	4.79	0.00	0.00	100.00	0.00	0.00	0.00
*Amphicyon giganteus*	2	112.75	0.25	19.00	1.50	5.93	0.00	0.00	100.00	0.00	100.00	0.00
*Percrocuta miocenica*	3	115.00	1.53	7.17	0.60	16.05	0.00	100.00	0.00	0.00	66.66	100.00
*Hemicyon goeriachensis*	9	93.33	0.66	12.50	0.35	7.47	0.00	11.11	88.88	44.44	77.77	0.00

Note: N, sample size; SP, average amount of small pits; SE-SP, standard error of small pits; LP, average amount of large pits; SE-LP, standard error of large pits; SP/LP, small pits/large pits ratio; %F, percentage of individuals per taxon with fine scratches; %C, percentage of individuals per taxon with coarse and hypercoarse scratches; %M, percentage of individuals per taxon with both fine and coarse scratches; %PP, percentage of individuals per taxon with puncture pits; %G, percentage of individuals per taxon with gouges; %0–8 LP, percentage of individuals per taxon with 0–8 large pits

**Table 4:** Mesowear scores (MWS) and mesowear angles (MW angle) of fossil ungulates and proboscideans from Gračanica

	**Inventory number**	**Family**	**Taxon**	**Element**	**Sin/Dex**	**MWS**
**Ungulata**	2013/0003/0002a	Rhinocerotidae	*Plesiaceratherium balkanicus*	M2	Sin	1.5
	2013/0004/0006	Rhinocerotidae	*Brachypotherium brachypus*	M2	Sin	1.5
	2013/0005/0008	Equidae	*Anchitherium ezquerrae*	P4	Dex	2.0
	2013/0006/0005a	Equidae	*Anchitherium hippoides*	M2–3	Sin	1.5
	2013/0006/0005b	Equidae	*Anchitherium hippoides*	M1	Sin	1.5
	2013/0006/0005c	Equidae	*Anchitherium hippoides*	M1–2	Dex	1.5
	2013/0006/0007	Equidae	*Anchitherium hippoides*	M1–2	Sin	1.5
	2013/0006/0008	Equidae	*Anchitherium hippoides*	P3–4	Sin	1.5
	2013/0007/0001	Palaeomerycidae	Palaeomerycidae indet.	M2/3	Sin	2.0
	2013/0011/0003	Bovidae	*Eotragus* ?*clavatus*	m3	Dex	2.0
	2013/0011/0003	Bovidae	*Eotragus* ?*clavatus*	m3	Sin	2.0
	2014/0082/0005	Chalicotheriidae	*Anisodon* cf. *grande*	M1	Sin	3.0
	2014/0082/0006	Chalicotheriidae	*Anisodon* cf. *grande*	M3	Sin	2.0
	2014/0082/0007	Chalicotheriidae	*Anisodon* cf. *grande*	M1	Sin	3.0
	**Inventory number**	**Family**	**Taxon**	**Element**	**Sin/Dex**	**MW angle**
**Proboscidea**	2013/0002/0003	Gomphotheriidae	*Gomphotherium angustidens*	M3	Dex	105.10
	2013/0002/0001	Gomphotheriidae	*Gomphotherium angustidens*	m3	Sin	104.13
	2012/0153/0001c	Deinotheriidae	*Prodeinotherium bavaricum*	m2	Dex	87.20
	2012/0153/0001d	Deinotheriidae	*Prodeinotherium bavaricum*	m3	Dex	93.00

Fossil material of ruminants from Gračanica is scarce (Aiglstorfer and Mayda in press, this issue). Therefore, due to the small sample size, the results presented herein should be taken with caution. The microwear signal of the ruminants is very consistent, showing a typical browsing behaviour. As depicted in Fig. 3.a, all ruminants from Gračanica demonstrate a relatively similar number of scratches, and thus, are distributed vertically within the browsing morphospace, due to their differences in the total number of pits. *Dorcatherium vindebonense* exhibits the lowest number of pits, as well as a relatively high amount of puncture pits, especially in regard to the other fossil ruminants. This taxon also possesses a moderate number of scratches, while gouges are completely absent. *Eotragus?clavatus* displays the highest amount of pits among the ruminants. However, the sample exhibits an insignificant amount of puncture pits and no gouges. Between the two morphospaces occupied by the above-mentioned taxa, *Giraffokeryx* sp. and Palaeomerycidae indet. are clustering, with the latter exhibiting a slightly larger number of pits and an insignificantly smaller number of scratches. However, both *Giraffokeryx* sp. and Palaeomerycidae indet. show a similar puncture pit count, as well as the presence of gouges. Overall, there is an almost complete absence of hypercoarse scratches, a small fluctuation of coarse scratches (0–3), and a generally moderate amount of fine scratches (9–14). This implies a general consumption of clean food, with a meagre to almost absent exogenous dust and grit incorporated in the feeding process (Semprebon and Rivals 2007). The general absence of molars allowed only two ruminant taxa to be included in the mesowear analysis: *Eotragus?clavatus* and Palaeomerycidae indet. Both taxa exhibit MWS of 2.0, fitting within the general range of extant browsing ungulates (*Litocranius walleri*, *Naemorhedus crispus*, *Tragelaphus strepsiceros*; Fig. 4).

Suids are one of the most well-represented groups at Gračanica. The available material was assigned to three different species: *Bunolistriodon latidens*, *Choeromorus lemuroides,* and *Conohyus simorrensis* (van der Made in press, this issue). *Bunolistriodon* exhibits on average a relatively high number of pits, but a low amount of puncture pits, as well as a moderate number of scratches. *Choeromorus* shows the highest number of scratches and pits among all suids, as well as the highest amount of puncture pits. *Conohyus* exhibits the lowest average number of scratches and pits, but a moderate number of puncture pits. All fossil suid specimens exhibited heavy gouging, but showcase a moderate number of scratches and very little or absent hyper-coarse scratches. All three suid taxa plot within the area of generalists, with *C. simorrensis* leaning towards the browsing morphospace (Fig. 3.b). Due to their dental morphology, suids were excluded from the mesowear analysis.

Fossil Rhinocerotidae constitute one of the most abundant groups from Gračanica, with four different taxa described: *Brachypotherium brachypus*, *Hispanotherium* cf. *matritense*, *Lartetotherium sansaniense*, and *Plesiaceratherium balkanicum* (Becker and Tissier in press, this issue). The majority of the dental material revealed a clear dental microwear signal. However, due to partly fragmentary apices of most molars, only two specimens were included in the dental mesowear analysis. The microwear data of the rhinocerotids suggests a browsing to mixed-feeding behaviour. *Brachypotherium brachypus* and *Plesiaceratherium balkanicum* exhibit a significant overlapping and occupy the same morphospace, revealing a high number of pits and a relatively low number of scratches (Fig. 3.c). *Lartetotherium sansaniense* displays the highest number of pits and a relatively high number of mostly fine scratches. *Hispanotherium* cf. *matritense* reveals a clearer mixed-feeding pattern with the average amount of scratches being 20.0. Additionally, the number of pits is still relatively high, approximately at the same level as *P. balkanicum*. The two molars included in the mesowear analysis derive from *P. balkanicum* (NHMW 2013/0003/0002a) and *B. brachypus* (NHMW 2013/0004/0006). Both molars have a MWS of 1.5, revealing a classic browsing tendency (Table 4; Fig. 4.b).

Equids are represented by two taxa: *Anchitherium ezquerrae* and *Anchitherium hippoides* (Becker et al. in press, this issue). The two taxa exhibit a significant overlapping, with *A. hippoides* generally plotting closer to the browsing morphospace (Fig. 3.d). Both equids plot between the browsing and grazing morphospaces, demonstrating a small amount of puncture pits, while gouging is more prominent in *A. ezquerrae*. Following Kaiser and Solounias (2003), we include P4, M1, M2, and M3 in our mesowear analysis for the fossil equids. The results reveal a mostly browsing signal with a MWS of 1.5 for *A. hippoides* and 2.0 for *A. ezquerrae* (Table 4; Fig. 4.b). However, the latter is represented only by one tooth and therefore the results should be considered with caution.

The fossil material of Chalicotheriidae from Gračanica revealed the presence of *Anisodon* cf. *grande* (Coombs and Göhlich in press, this issue). Only seven adult teeth were recovered from which none revealed a clear dental microwear signal, probably due to taphonomic alterations. However, the decent preservation and moderate wear of the permanent molars allowed for the application of dental mesowear analysis. The taxon exhibits an average MWS of 2.6, fitting close to the general range of mixed-feeding taxa (Table 4; Fig. 4.b).

The fossil proboscideans revealed the presence of four taxa: *Prodeinotherium bavaricum*, *Gomphotherium angustidens*, cf. *Protanancus* sp., and cf. *Gomphotherium subtapiroideum*. The two latter taxa are only represented by deciduous dentition and therefore were not included in either the microwear or the mesowear analysis. *Prodeinotherium bavaricum* possesses a high number of pits and a relatively small number of scratches, plotting close to the browsing morphospace (Fig. 3.e). *Gomphotherium angustidens*, even though it shows an overall browsing-like microwear signal, demonstrates a significantly lower number of pits and a larger number of scratches than *P. bavaricum*. Both taxa exhibit few small puncture pits and neither large puncture pits nor gouges. Both of the Gračanica proboscideans have mesowear angles indicating strongly browse-based diets, although due to the very low sample size, they might not represent the total extent of their dietary range, especially in the case of *Gomphotherium angustidens*.

**Fig. 5:**
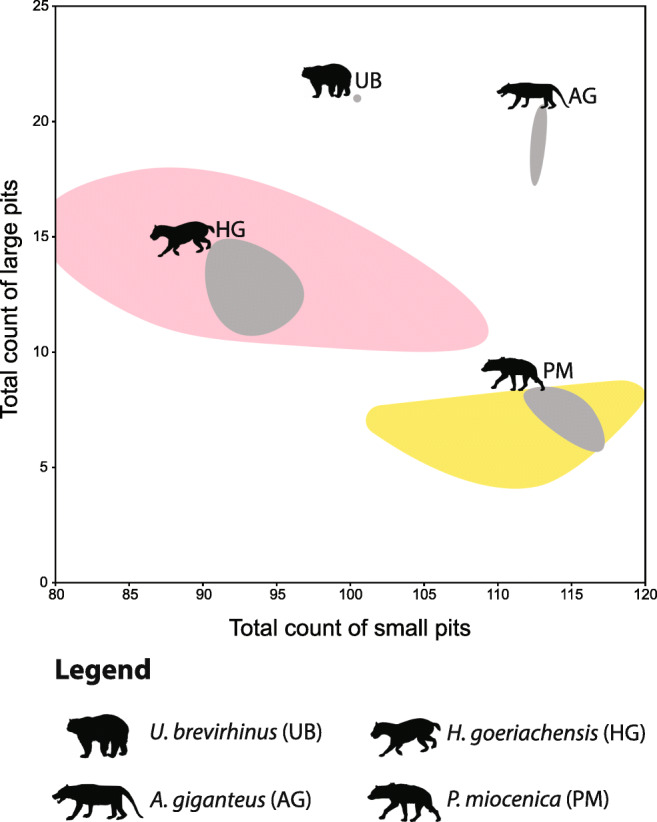
Dental microwear results of fossil carnivores from Gračanica, plotted on Total count of small pits versus Total count of large pits scatter plot. Grey ellipses represent the distribution of each fossil taxon. Yellow highlighted area represents the morphospace of extant bone crushing hypercarnivores (*Hyaena hyaena*). Pink highlighted area represents the morphospace of extant meat specialist hypercarnivores (*Acinonyx jubatus*) (based on Bastl et al. 2012: Xafis et al. 2017). For identification of taxa consult the symbol legend. Animal silhouettes from PhyloPic.org

The fossil collection from Gračanica also revealed the presence of a small Carnivora assemblage, including *Amphicyon giganteus*, *Hemicyon goeriachensis*, *Ursavus brevirhinus*, *Percrocuta miocenica*, and Mustelidae indet. (Bastl et al. in press, this issue). *Hemicyon goeriachensis* constitutes the most abundant taxon, while *Ursavus brevirhinus* and Mustelidae indet. are represented by one specimen each. The latter did not exhibit any dental microwear and was therefore excluded from the analysis. *Ursavus brevirhinus* and *Amphicyon giganteus* exhibit a very similar microwear signal. Both taxa show a notably high number of large pits as well as a relatively high number of small pits, plotting the closest to the cheetah (*Acinonyx jubatus*) morphospace. Similarly, *H. goeriachensis* shows an average of 93.3 small pits and 12.5 large pits, plotting within the cheetah morphospace. *Percrocuta miocenica* shows a typical hyaena microwear signal, plotting the closest to the striped hyaena (*Hyaena hyaena*) (Fig. 5).

## Discussion

Based on the potential modern analogues included in the extant comparative datasets, the fossil herbivores from Gračanica exhibit browsing and mixed-feeding dietary behaviours, while pure grazers are completely absent. Taxa categorized as browsers are all ruminants and proboscideans, as well as *P. balkanicum* and *B. brachypus*. Equids, suids, and the rhinocerotids *L. sansaniense* and *Hispanotherium* cf. *matritense* demonstrate mixed feeding tendencies. In detail, the overall microwear signal in combination with the low MWS reveals a browsing behaviour of all ruminant taxa recovered from Gračanica. There are only a few studies regarding the dietary ecology of *Dorcatherium*. Therefore, their exact dietary adaptation and evolution is far from resolved. Previous research based on stable isotopic measurements and dental wear analyses revealed that the diet of *Dorcatherium* may fluctuate from leaf browsing to fruit- or even grass-dominated mixed feeding behaviour (Kaiser and Rössner 2007; Merceron 2009; Ungar et al. 2012; Aiglstorfer et al. 2014; Eastham et al. 2016). However, *Dorcatherium vindebonense* is believed to have been a mixed feeding taxon. More specifically, dental microwear analysis of *D. vindebonense* from the Styrian and Vienna basins revealed a high number of scratches (Merceron et al. 2012). Isotopic measurements on specimens from the Göriach locality (Styria, Austria) demonstrated δ^18^O values suggesting that *Dorcatherium* could occupy different ecological niches during the middle Miocene (Aiglstorfer et al. 2014). Palaeoenvironmental reconstruction of the middle Miocene of Rusinga Island and Songhor (Kenya, East Africa), based on microwear texture analysis of four tragulid taxa, show that all encountered tragulids were mixed feeders and two of them were similar to extant grazers (Ungar et al. 2012). This observation led the authors to suggest that the fossil tragulids reflect a more diverse dietary adaptation than observed in contemporary taxa. Our results showcase a browsing signal for *D. vindebonense* from Gračanica. However, the specimens included in the study demonstrate a moderate number of puncture pits, as well as a low number of coarse or hypercoarse scratches. This combination of features reflects a typical frugivorous signal, with a high leaf-browsing tendency. Further studies on the dietary preferences and adaptation of fossil European tragulids would be essential to reveal whether a behaviour similar to that of the East African Miocene Tragulidae applies to the Miocene tragulids of Europe.

Previous palaeoecological investigations of *Eotragus* from Sansan (France), the Calatayud-Daroca Basin (Spain) and Göriach (Austria), reveal a consistent browsing microwear signal (Solounias and Moelleken 1992, 1993; DeMiguel et al. 2011; Merceron et al. 2012). These analyses were established by utilising different methodologies than in the present study, therefore, a direct comparison of dental wear values is not possible. Regardless, *Eotragus* from Gračanica exhibits a clear browsing microwear signal, in agreement with the above-mentioned palaeodietary studies.

Previous analyses (e.g. dental microwear, dental mesowear, and stable isotopes) have revealed that *Giraffokeryx* had diverse dietary habits, ranging from browsing to seasonal mixed feeding (Quade et al. 1995; Solounias et al. 2000; Tariq and Jahan 2014). In detail, Solounias et al. (2000) and Tariq and Jahan (2014) revealed a browse-dominated mixed-feeding diet for *Giraffokeryx punjabiensis* from Pakistan. However, a study on the middle Miocene Paşalar palaeocommunity, located in the eastern part of the Dinarides-Anatolian Island, showed that *G. punjabiensis* was a canopy browser, feeding predominantly on C3 vegetation (Quade et al. 1995). Our results indicate a browsing signal for *Giraffokeryx* sp. from Gračanica, but not similar to an extreme browser, such as the giraffe and/or the gerenuk. Thus, the Gračanica and the Paşalar populations seem to have had similar dietary traits, suggesting uniform dietary preferences for *Giraffokeryx* throughout the Dinarides-Anatolian Island.

The Palaeomerycidae comprises mainly browsing taxa with brachydont to mesodont dentition (Semprebon et al. 2004b; Prothero and Liter 2007; Janis 2008). Palaeodietary reconstructions of the family have been carried out several times using various proxies, such as the premaxillary shape (Solounias and Moelleken 1993), stable isotopes (MacFadden and Cerling 1996; Kita 2011), as well as dental microwear and mesowear analyses (Semprebon et al. 2004b; Kaiser and Rössner 2007; Merceron et al. 2012; Fraser and Theodor 2013). Dental wear analysis of palaeomerycids from North America showed that the family was dominated by browsing taxa, with few derived species demonstrating higher tooth crowns and mixed-feeding tendencies (Semprebon et al. 2004b). Using the same proxies on European palaeomerycids, Kaiser and Rössner (2007) presented a dietary pattern very similar to that of the North American taxa. In both cases, dietary shifts were driven by environmental changes, such as aridity, which drove palaeomerycids to feed on more abrasive nutriments (Semprebon et al. 2004b; Kaiser and Rössner 2007). Palaeomerycidae indet. from Gračanica reveals a typical browsing behaviour. Collectively, the samples included in the analysis show similar values to the gerenuk, which is considered an extreme browser. However, all teeth exhibit a fair number of large pits, as well as a small amount of puncture pits. Therefore, we assume a fair amount of fruit and/or seed intake incorporated in the diet of Palaeomerycidae indet. from Gračanica.

Past research on the diets of middle Miocene Suidae is rather scarce and therefore, our data will contribute significantly to the resolution of the palaeoecology of the family. *Conohyus* has been considered a mixed-feeder with significant amount of rooting incorporated in their diet (Aiglstorfer et al. 2014) or even as an omnivore (Eastham et al. 2017). It has also been shown that this taxon inhabited open environments, with a predominantly C4 vegetation (Quade et al. 1995; Domingo et al. 2012). *Conohyus* from Gračanica exhibits a moderate number of scratches, with 40% of the sample having 0–17 scratches, suggesting an important amount of soft tissue in their diet (Semprebon et al. 2016) (Table 2). In addition, the taxon shows a moderate number of coarse scratches, as well as gouges which is well fitted to a “dirty browsing” behaviour. Conclusively, our data are in agreement with Aiglstorfer et al. (2014) and we propose an opportunistic diet with a lot of dirt-covered soft tissues, such as roots, for *C. simorrensis* from Gračanica.

Even though listriodonts, based on their dental features, have been considered grazers (van der Made 2003), there is a complete absence of research on the dietary ecology of *Bunolistriodon*. Our data show a separation of *B. latidens* from *C. simorrensis*, with the former showing a significantly higher average number of both scratches and pits (Table 2). The complete sample of *Bunolistriodon* from Gračanica demonstrated more than 17 scratches and therefore, a constant intake of highly abrasive items is suggested (Table 2). In addition, gouging is constant in all teeth included in the analysis, while puncture pits are very few to absent. Thus, our results suggest that *B. latidens* from Gračanica had a seasonal grass-dominated opportunistic diet.

Generally, middle Miocene Taucanaminae are considered leaf-browsing taxa, based on their tooth morphology (Agustí 2007). However, dental wear data are completely absent. Our results place *Choeromorus lemuroides* quite far from the browsing morphospace (Fig. 3.b). In addition, 100% of the sample exhibited more than 17 scratches, revealing a high intake of abrasive particles (Table 2). The taxon also shows a significant amount of puncture pits, as well as gouges. Therefore, we suggest for *C. lemuroides* a mixed-feeding diet similar to *B. latidens*, possibly with a higher intake of fruits and seeds.

Previous studies on the palaeoecology of *Lartetotherium* revealed a possibly opportunistic dietary behaviour. In detail, Aiglstorfer et al. (2014) concluded on an open environment feeding for *L. sansaniense* from Gratkorn, while Becker and Tissier (in press, this issue) supported a mixed environment for the taxon, which possibly varied from more open to closed spaces. Our data are in agreement with the previous studies, since the sample of *L. sansaniense* from Gračanica plots within the mixed-feeding morphospace (Fig. 3.c). In addition, 50% of the sample scored between 0 and 17 scratches, which eliminates the possibility of a clear browsing behaviour for *Lartetotherium* (Table 2). The low amount of puncture pits, in combination with the low number of coarse scratches and the absence of hypercoarse scratches, does not support regular fruit-dominated browsing habits, even though seed intake was most likely occasional. Therefore, we suggest that *L. sansaniense* was feeding in somewhat open environment.

The ecology of *Brachypotherium* has been a matter of debate. Due to the interesting skeletal structure of the taxon, a hippopotamus-like lifestyle has been assumed (Geraads et al. 2003; Geraads and Spassov 2009). However, the majority of previous palaeoecological studies on *Brachypotherium* indicate a non-selective browsing behaviour within a closed-environment (Sehgal and Nanda 2002; Tütken et al. 2006; Heissig 2012; Aiglstorfer et al. 2014). The dental microwear signal of *B. brachypus* from Gračanica reveals a general browsing signal. The total number of scratches fluctuates between 10.0 and 15.5, never exceeding 17 scratches (Table 2). Nevertheless, the large percentage of large pits, as well as the significant gouging could indicate a “dirty-browsing” behaviour. Such a dietary habit implies browsing close to the soil surface, allowing higher amount of dust and grit into the mouth. In fact, postcranial morphology suggests a close-to-the-ground browsing habit due to brachypody and a low head posture (Becker and Tissier in press, this issue), which would serve a “dirty browsing” diet.

*Plesiaceratherium balkanicum* is a new species described from Gračanica by Becker and Tissier (in press, this issue). Therefore, our results constitute the first palaeoecological study of the taxon. Past studies on *Plesiaceratherium*, which were mostly based on isotopic measurements, have shown that the genus inhabited both open and closed environments (Tütken et al. 2006; Tütken and Vennemann 2009; Aiglstorfer et al. 2014; Becker and Tissier in press, this issue). Our microwear and mesowear results show a significant overlapping between *P. balkanicum* and *B. brachypus*, with both taxa reflecting a strong browsing tendency (Fig. 3.c; Fig. 4.b). In addition, all specimens of both taxa exhibited a low scratch range, justifying a strong browsing signal (Semprebon et al. 2016). *Plesiaceratherium*, however, does not exhibit any gouging, while most teeth revealed the presence of large puncture pits. Therefore, a “dirty browsing” behaviour cannot be assumed. Similar to *Brachypotherium*, *Plesiaceratherium* does not possess a significant amount of hyper-coarse scratches. Thus, even though the two taxa seem to have occupied the same ecological niche, a “cleaner” browsing behaviour with a relatively regular intake of fleshy fruit and seeds is proposed for *P. balkanicum*, as opposed to the “dirty browsing” habits of *B. brachypus*.

Previous research on the feeding ecology of *Hispanotherium* showed that the taxon fluctuated from mixed-feeder to grazer, always occupying a rather open, likely drier, environment (Fortelius et al. 2002; Domingo et al. 2012; Becker and Tissier in press, this issue). However, dental wear data for *Hispanotherium* are completely absent from the literature. Our results support previous assumptions since *H.* cf. *matritense* exhibits a relatively high number of scratches, plotting closer to the grazing morphospace than the other rhinocerotids (Fig. 3.c). All specimens demonstrate more than 17 scratches, which explains the high amount of abrasives incorporated in the diet of *H*. cf. *matritense* (Table 2). At the same time, the taxon reveals an average of 59.13 pits, which is much higher than the amount a regular grazer demonstrates. Due to the absence of gouges and hyper-coarse scratches, it is unlikely that *Hispanotherium* had a “dirty” eating behaviour. The presence of both small and large puncture pits demonstrates a potential occasional fruit consumption. Thus, we conclude that *Hispanotherium* cf. *matritense* from Gračanica was a grass-dominated mixed-feeder with an occasional fruit intake.

The family Equidae is represented at Gračanica with two species, the dietary ecology of which has never been studied in the past. However, previous research on different species of *Anchitherium* have revealed that the genus could show dietary patterns low in abrasives (Mihlbachler et al. 2011), be adaptable generalists (Tütken and Vennemann 2009; Eronen et al. 2010), “dirty browsers” (Kaiser 2009), or forest dwelling browsers (MacFadden 2009). Our microwear results reveal an initially mixed-feeding signal, with the two species showing significant overlapping (Fig. 3.d). The mesowear results show a MWS of 1.5 for *A. hippoides* and 2.0 for *A. ezquerrae*, with the latter being represented by only one specimen (Table 4; Fig. 4.b). The complete sample of *A. hippoides* demonstrated 0–17 scratches, while only 44.44% of *A. ezquerrae* plotted within this range (Table 2). In addition, more than 50% of *A. ezquerrae* specimens exhibit gouging, while in *A. hippoides*, gouging is almost absent. Both display few small puncture pits, with *A. ezquerrae* having a slightly higher average number of puncture pits. Thus, even though the two species seem to occupy similar ecological niches, our results suggest a higher intake of abrasives for *A. ezquerrae*. A “dirty browsing” behaviour, similar to *A. aurelianense* from Sandelzhausen (Kaiser 2009), is suggested for *A. ezquerrae*. On the other hand, *A. hippoides* is believed to be a leaf-dominated mixed feeder with some seasonal fleshy fruit consumption.

The palaeoecology of Chalicotheriidae has been investigated in the past by utilising dental wear analysis, showing that *Anisodon* was mainly a browser with a significant intake of abrasives such as bark, fruit, and seeds (Schulz et al. 2007; Schulz and Fahlke 2009; Semprebon et al. 2011). However, Fahlke et al. (2013) showed that towards the late Miocene, *Anisodon* exhibits a browsing dental wear signal with no considerable amount of abrasive particles. As mentioned above, the dental material of *A.* cf. *grande* from Gračanica preserved no microwear scarification and it was only included in our mesowear analysis. The results reveal a MWS of 2.6 which indicates a mixed-feeding diet (Fig. 4.b). Therefore, based on our results and previous studies on middle Miocene material, we suggest a browsing diet for *A.* cf. *grande* from Gračanica, with a possible intake of fruit, seeds, and bark, which are responsible for the mixed-feeding mesowear signal.

Proboscideans are represented at Gračanica by four different taxa (Göhlich in press, this issue), from which, as mentioned above, only *Prodeinotherium bavaricum* and *Gomphotherium angustidens* were included in our dental wear study. The family Deinotheriidae is known to include species, which were exclusively leaf browsers (Harris and Cerling 1996; Calandra et al. 2008) feeding on C3 vegetation (Cerling et al. 1997, 1999; Aiglstorfer et al. 2014). The family Gomphotheriidae, however, includes generalists which had a higher amount of abrasives incorporated in their diets, but still fed mostly, or exclusively, on C3 vegetation (Quade et al. 1995; Tütken and Vennemann 2009; Domingo et al. 2009, 2012; Aiglstorfer et al. 2014). Our dental microwear results indicate a clear browsing signal for *Prodeinotherium bavaricum*. A portion of the sample seems to plot outside the browsing morphospace (Fig. 3.e), which could be explained by the seasonal and/or occasional consumption of non-grazing abrasive items, such as fleshy fruit pits. This can also be justified by the moderate amount of large pits and the small number of puncture pits. *Gomphotherium angustidens* also reveals a strong leaf browsing signal. The average number of total scratches, as well as the higher average amount of course scratches, exhibits the presence of more abrasives in their diet in comparison to *P. bavaricum*. Both taxa, however, have 100% of their sample with 0–17 scratches justifying a strong leaf browsing behaviour (Table 2).

**Fig. 6:**
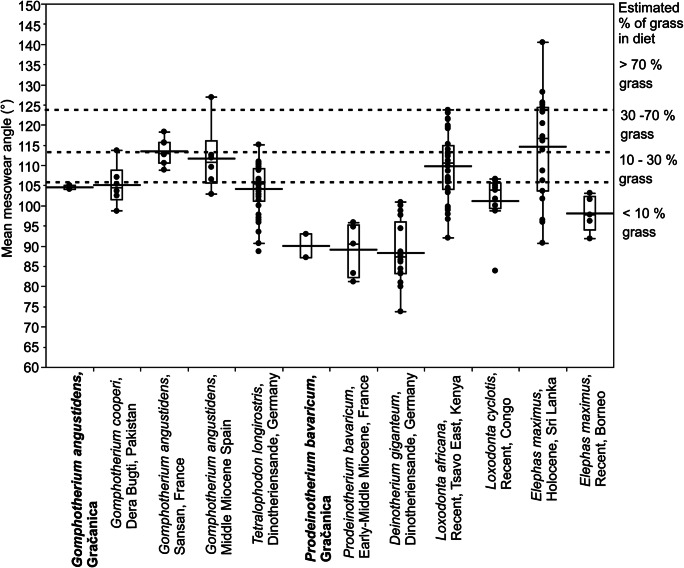
Box plots of the Gračanica proboscidean mesowear angles compared with the mean mesowear angles of other Miocene and present proboscideans. The estimated proportion of grass in the diet is based on the comparison of C4 grasses in diet (based on stable carbon isotope results) and population-level mean mesowear angles of tropical proboscidean populations (Saarinen et al. 2015). See Table 5 for pairwise Wilcoxon test comparisons of the mesowear angles

The mesowear angles of the proboscidean taxa are in agreement with the microwear results. In detail, the *Prodeinotherium bavaricum* molars show characteristic sharp, steeply angled facets with resulting mean mesowear angles very close to 90°, indicating purely browsing diet (Fig. 6). The approximately 90° mean mesowear angle is statistically indistinguishable from the mean mesowear angles of the other deinothere samples included here for comparison (Fig. 6; Table 5), and indicates similar diet despite the low sample size. The mesowear angle measurements for each loph/lophid of each proboscidean molar from Gračanica are shown in the Online Resource 3.

**Table 5:** Pairwise Wilcoxon test comparisons of the mean mesowear angles of the Gračanica proboscideans with other Miocene and extant proboscidean populations

**Population**	**Population**	**Score Mean Difference**	**Std Err Dif**	***Z***	***p***
*Deinotherium giganteum*, Eppelsheim	*Gomphotherium angustidens*, Gračanica	-9.20	4.20	-2.20	0.03
*Gomphotherium angustidens*, Sansan	*Gomphotherium angustidens*, Gračanica	4.20	2.20	1.90	0.06
*Prodeinotherium bavaricum*, France	*Gomphotherium angustidens*, Gračanica	-3.20	1.80	-1.70	0.08
*Prodeinotherium bavaricum*, Gračanica	*Gomphotherium angustidens*, Gračanica	-1.50	1.30	-1.20	0.25
*Loxodonta africana*, Tsavo East (Kenya)	*Gomphotherium angustidens*, Gračanica	7.70	7.30	1.10	0.29
*Elephas maximus*, Sri Lanka	*Gomphotherium angustidens*, Gračanica	4.70	4.60	1.00	0.31
*Loxodonta cyclotis*, Congo	*Gomphotherium angustidens*, Gračanica	-1.50	2.60	-0.60	0.56
*Gomphotherium cooperi*, Dera Bugti	*Gomphotherium angustidens*, Gračanica	0.00	2.00	0.00	1.00
*Gomphotherium angustidens*, Gračanica	*Elephas maximus*, Borneo	3.20	1.80	1.70	0.08
*Tetralophodon longirostris*, Dinotheriensande	*Gomphotherium angustidens*, Gračanica	2.10	7.70	0.30	0.78
*Gomphotherium angustidens*, Gračanica	*Gomphotherium angustidens*, mid Miocene, Spain	-2.30	2.00	-1.20	0.24
*Tetralophodon longirostris*, Dinotheriensande	*Prodeinotherium bavaricum*, Gračanica	16.70	7.70	2.20	0.03
*Loxodonta africana*, Tsavo East (Kenya)	*Prodeinotherium bavaricum*, Gračanica	16.20	7.30	2.20	0.03
*Elephas maximus*, Sri Lanka	*Prodeinotherium bavaricum*, Gračanica	9.70	4.60	2.10	0.04
*Prodeinotherium bavaricum*, Gračanica	*Elephas maximus*, Borneo	-2.50	1.80	-1.40	0.18
*Prodeinotherium bavaricum*, Gračanica	*Gomphotherium angustidens*, mid Miocene, Spain	-3.70	2.00	-1.80	0.07
*Loxodonta cyclotis*, Congo	*Prodeinotherium bavaricum*, Gračanica	4.00	2.60	1.50	0.13
*Deinotherium giganteum*, Eppelsheim	*Prodeinotherium bavaricum*, Gračanica	-1.40	4.20	-0.30	0.74
*Prodeinotherium bavaricum*, France	*Prodeinotherium bavaricum*, Gračanica	0.00	1.80	0.00	1.00
*Prodeinotherium bavaricum*, Gračanica	*Gomphotherium angustidens*, Sansan	-4.20	2.20	-1.90	0.06
*Prodeinotherium bavaricum*, Gračanica	*Gomphotherium cooperi*, Dera Bugti	-3.70	2.00	-1.80	0.07
*Prodeinotherium bavaricum*, Gračanica	*Gomphotherium angustidens*, Gračanica	-1.50	1.30	-1.20	0.25

The two *Gomphotherium angustidens* molars from Gračanica show relatively steeply angled wear facets and deep worn dentine valleys, resulting in mean mesowear angles of 104°–105°, indicating browsing or heavily browse-dominated diet (Fig. 6). The mean mesowear angles of the Gračanica *G. angustidens* are almost identical to the mean mesowear of the larger sample of *G. cooperi* from the early Miocene of Dera Bugti, Pakistan, and *Tetralophodon longirostris* from the Vallesian (early late Miocene) of Dinotheriensande, Germany, and they are also very close to the average mesowear signal of the extant African forest elephant (*Loxodonta cyclotis*) from Congo, and the rainforest population of the Asian elephant (*Elephas maximus*) from Borneo (Fig. 6).

The mesowear signal of *Gomphotherium angustidens* from Gračanica differs considerably from the mixed-feeding mesowear signal of *G. angustidens* from Sansan, France (Fig. 6). The former one fits within the range, but is considerably more browse-dominated than the means of the highly variable, on average mixed-feeding mesowear signals of *G. angustidens* from the middle Miocene of Spain, the extant African savanna elephant (*Loxodonta africana*) from Tsavo East, Kenya, and the Asian elephant (*Elephas maximus*) from Sri Lanka (Fig. 6). However, these differences mostly do not appear statistically significant in the means comparison by the Wilcoxon test, because of the low sample size of the Gračanica *Gomphotherium* (Table 5). Nonetheless, the mesowear signal of *Prodeinotherium bavaricum* from Gračanica is significantly different from the grass-rich mixed-feeding signal of *Elephas maximus* from Sri Lanka, while it does not significantly differ from the browsing mesowear signal of *Elephas maximus* from Borneo (Table 5).

The huge diversity of large mammalian herbivores at Gračanica demonstrates the ability of multiple herbivore taxa to coexist within the same community, achieved by partitioning the available food resources. Niche partitioning has previously been recognised in some mammalian palaeocommunities (Kaiser and Rössner 2007; Merceron et al. 2007; Calandra et al. 2008; Rivals and Lister 2016; Aiglstorfer and Semprebon 2019). As depicted in Fig. 3, the vast majority of the Gračanica herbivores are clearly separated from each other, confirming a niche segregation in the Gračanica palaeocommunity. For example, even though the ruminant assemblage consists exclusively of browsing taxa (Fig. 3.a), they clearly fed on different resources. Similarly, the browsing Rhinocerotidae exhibit a large overlapping (Fig. 3c), but their microwear values show a separation. *Brachypotherium brachypus* was most likely a “dirty browser” and *Plesiaceratherium balkanicum* was more of a leaf- and fruit-dominated browser. The fossil Equidae reveal a similar pattern, suggesting that *A. ezquerrae* was a “dirty browser” and *A. hippoides* was a leaf-dominated mixed feeder. *Prodeinotherium bavaricum* was the largest browser at Gračanica, feeding in higher levels of vegetation than other leaf browsers. The niche partitioning between the proboscideans *Prodeinotherium* and *Gomphotherium* is rather obvious, since *G. angustidens* incorporated a higher amount of abrasives in its diet, than the ever-browsing Deinotheriidae (Calandra et al. 2008). The Suidea, as well as *L. sansaniense* and *H.* aff. *maritense*, probably alternated between closed and open area, which is reflected by their opportunistic behaviour. These taxa most likely fed on a variety of resources, including roots, fruits, seeds, and grasses as well as other herbaceous monocots.

The fossil collection of Carnivora from Gračanica revealed the presence of hypocarnivorous, mesocarnivorous, as well as hypercarnivorous taxa (Bastl et al. in press, this issue). Despite the small sample size, our results show a clear separation of dietary traits within the Gračanica carnivores and allows for a clearer palaeoecological interpretation.

To date, there are no studies on the dental microwear of *Ursavus*. The genus, as the vast majority of Ursoidea, has been considered as hypocarnivorous/omnivorous (Ginsburg 1999; Kostopoulos and Vasileiadou 2006; Nagel and Koufos 2009; Koufos and Konidaris 2011; Pappa et al. 2019). The microwear scores on *U. brevirhinus*, even though they plot relatively close to the cheetah morphospace, reveal an unusually high number of large pits (Fig. 5). In addition, *Ancinonyx jubatus* exhibits a higher number of scratches. Due to the very restricted sample size (single tooth), further study is suggested to give a clearer picture on the dietary habits of *Ursavus*. However, based on the few data we collected in combination with the tooth morphology, we agree with the general assumption of an omnivorous diet. We attribute the high number of large pits to a significant fruit intake.

The family Amphicyonidae includes predominantly mesocarnivores and has only a few specialised hypercarnivores (Van Valkenburgh 1991; Goillot et al. 2009). *Amphicyon giganteus* is a relatively common European species that has been considered a bone-crushing carnivore (Viranta 1996; Ginsburg 1999). However, the genus shows a relatively large variation in dietary habits, since certain species of the North American *Amphicyon* are considered to be omnivorous (Hunt 2003). Goillot et al. (2009) were the first to conduct a dental microwear analysis in Amphicyonidae (*Amphicyon major* and *Pseudocyon sansaniensis*) revealing an omnivorous microwear signal for *A. major*. Much the same as *Ursavus*, *A. giganteus* from Gračanica shows a very high number of large pits as well as an intermediate number of scratches. Our results correlate well with the conclusions of the previous study. More specifically, all the specimens of *A. giganteus* possess more than eight large pits (Table 3), much like the extant meat specialists, some piscivores, or mixed feeders (Xafis et al. 2017). Therefore, taking into consideration previous analyses and suggestions, we consider *Amphicyon giganteus* to have had the same omnivorous/mixed-feeding dietary habit as *Amphicyon major*. Subsequently, the microwear of *A. giganteus* differs excessively from those of bone crushing taxa, such as the modern hyaenas, and thus, a bone-crushing diet is not considered for *A. giganteus* from Gračanica.

Hemicyonids constitute one of the ruling predatory European carnivore mammals from the early to late Miocene, when they gradually became extinct and were replaced by ursids (Abella 2014). To date, comprehensive palaeoecological analyses using dental wear proxies are absent. Nevertheless, previous studies based on morphometrical data and general morphological observations concluded on either a canid-like mesocarnivorous, or a lion or hyaena-like hypercarnivorous diet (Viranta 1996; Goswami et al. 2010; Abella 2014). *Hemicyon goeriachensis* is the most abundant carnivore from Gračanica, providing a descent sample size for our analysis (Table 3). As mentioned above, *H. goeriachensis* plots within the cheetah morphospace (Fig. 5). In addition, all specimens exhibit more than eight large pits (Table 3), placing the taxon among all other extant meat eaters (Xafis et al. 2017). The amount of large pits observed in *H. goeriachensis* eliminates any association with a hyaena-like bone-crushing eating habit. This in combination with the overall microwear signal, designates *H. goeriachensis* from Gračanica as a primarily meat eating hypercarnivore.

Percrocutidae is a poorly known group with an uncertain ecology (Van Valkenburgh 2007). Previous morphological studies assigned the diet of this extinct family to the bone-crushing diet of modern hyaenas (Deng and Tseng 2010; Stefen and Rensberger 1999) making percrocutids the earliest bone eaters (Qiu et al. 1988; Ginsburg 1999). Even though a thorough palaeoecological investigation of percrocutids has not yet been conducted, the dental microwear signal of *Percrocuta miocenica* from Gračanica agrees with the morphological data and thus, with the above-mentioned assumptions. Despite the small sample size (Table 3), the microwear signal is very consistent in separating *Percrocuta* from all other carnivores. More specifically, all teeth are clustering towards the bottom of the scatter plot, occupying the same morphospace as the striped hyaena (*Hyaena hyaena*) (Fig. 5). Additionally, all samples exhibit a low large-pit score (0–8 LP; Table 3), which is similar to the extant hyaenids (Xafis et al. 2017).

Studies on the palaeovegetation of Gračanica have revealed a plethora of taxa, recognised mostly by pollen (c. 60 pollen types; Jimémez-Moreno and Mandic in press, this issue) and some few macrofossil remains (Butzmann et al. in press, this issue). The fossil flora, composed of numerous different angiosperms as well as some gymnosperms, suggests a complexed and structured forest environment thriving under humid and subtropical conditions during the middle Miocene at Gračanica. Taxodioideae reveals the occurrence of a swampy lowland environment (Butzmann et al. in press, this issue), surrounded by mixed deciduous and evergreen broad-leaved forests at higher elevation. The existence of conifers such as *Pinus*, *Cathaya*, *Cedrus*, and *Picea* indicates mid- to high-altitude environments (Jimémez-Moreno and Mandic in press, this issue). This vegetation is in agreement with the dietary ecology of the mammal assemblage from Gračanica, since the diet preferences fluctuate between browsing and mixed feeding, with pure grazers being completely absent. The list of plant taxa reveals a mostly closed forest environment with occasional meadows, where grasses and other monocots occurred. Taxa such as *Bunolistriodon*, *Choeromorus*, and *Hispanotherium* were likely feeding in these meadows, where they were able to graze seasonally. The extended presence of herbaceous plant taxa, such as *Amaranthaceae*, *Apiaceae*, *Asteraceae*, and *Plantago* would have served as a great option for “dirty browsers” like *Conohyus*, *Brachypotherium*, and *A. ezquerrae*. Lastly, a large number of the fossil plant assemblage did bear edible fleshy fruits and/or seeds. These include among others *Rhus*, *Ilex*, *Microtropis*, *Nyssa*, and *Juglans*, which were seasonally providing for the fruit browsing mammals at Gračanica.

## Conclusions

The rich mammalian fossil assemblage recently unearthed from the coal mine deposits at Gračanica, Bosnia-Herzegovina, near Bugojno or in the Bugojno Basin allowed us to reconstruct the palaeodiets of this early middle Miocene mammalian fauna. This was the first attempt to reconstruct terrestrial palaeoenvironments of the DLS and the western part of the Dinarides-Anatolian Island, utilising a multiproxy dental wear analysis. We employed dental microwear and mesowear analysis on all available material. The results of both methodological approaches were consistent, revealing browsing tendencies for the majority of herbivorous taxa. We suggest a browsing diet for *Eotragus* ?*clavatus*, Palaeomerycidae indet., *Giraffokeryx* sp., *D. vindebonense*, *P. balkanicum*, *A. hippoides*, *A.* cf. *grande*, *P. bavaricum*, and *G. angustidens*, with a higher or lower intake of abrasives such as fruit pits and seeds. The species *C. simorrensis*, *B. brachypus*, and *A. ezquerrae* were possibly “dirty browsers”, with a higher intake of dust and grit in their food. Lastly, *B. latidens*, *C. lemuroides*, *H.* cf. *matritense*, and *L. sansaniense* are classified as mixed feeders, with either browsing or grazing tendencies.

Dental microwear analysis on the carnivore assemblage from Gračanica showed the presence of hypercarnivores, mesocarnivores, and hypocarnivores. *Ursavus brevirhinus* and *Amphicyon giganteus* are classified as omnivores/generalists. *Hemicyon goeriachensis* showed a signal of high meat intake, hypercarnivory, similar to the modern cheetah, and *Percrocuta miocenica* revealed a bone-crushing microwear signal similar to the modern striped hyaena.

The palaeodiets of the fossil mammals found at Gračanica suggest a closed forest environment, without significant open areas, since clear grazers are completely absent. This suggestion is validated by the fossil plant assemblage reconstructed by analysis of micro and macrofossils. The plant taxa reveal a humid subtropical climate, as well as the presence of swampy lake environments. The plant assemblage also reflects a possible food source for the herbivores of Gračanica. The dominant presence of broad-leaved fruit and seed-bearing plants along with a mixture of herbaceous taxa, grasses, and other monocots, justifies the coexistence of fruit and leaf browsers, “dirty browsers”, as well as grass-dominated generalists.

## Electronic supplementary material

ESM 1 (XLSX 77 kb)

ESM 2 (XLSX 5853 kb)

ESM 3 (XLSX 19 kb)

ESM 4 (XLSX 18 kb)

ESM 5 s (XLSX 12 kb)
